# Examining the applicability of hard data protection law on demographically identifiable information (DII): the case of humanitarian UAV/drone images in Malawi

**DOI:** 10.1186/s41018-025-00174-z

**Published:** 2025-07-21

**Authors:** Rogers Alunge Alunge Nnangsope

**Affiliations:** ITC/PGM, University of Twente, Drienerlolaan 5, Enschede, 7522 NB Netherlands

**Keywords:** Demographically identifiable information, Drones, Malawi, Data protection, Privacy, Malawi Data Protection Bill, Regulatory nudges, Humanitarian organisations, Disaster resilience

## Abstract

Research carried out in Northern Malawi concluded that protecting and ensuring responsible use of drone-collected data in disaster-risk areas should be left entirely to the humanitarian organisations collecting these data, with little participation expected of the affected residents, mostly because they will always be coerced into giving up their data in exchange for assistance. One way to guarantee this protection is by applying national rules of data protection law to these drone data processes in the country. However, aerial drone data (e.g. high-resolution images) of a community would usually be demographically identifiable information (DII) which, unlike personally identifiable information (PII), is not substantively regulated by contemporary hard data protection law, i.e. the 2023 Malawi Data Protection Bill (MDPB) which leaves drone DII without any binding regulatory framework and hence less legal protection.

Faced with this regulatory obstacle, this paper sets out to propose and evaluate methods through which the data processing principles and rights provided by the MDPB could nevertheless be applied to regulate drone DII collected and processed by humanitarian organisations in Malawi. First, it sought to establish the feasibility of their application among the humanitarian community: to this end, 20 semi-structured interviews were conducted with humanitarian officials operating in the country, with results showing that these officials largely believed the MDPB principles and rights could effectively govern their drone data processes. The paper then proposes some regulatory modifications or ‘nudges’ which, if adopted by Malawian regulators, could probe humanitarian organisations towards applying the MDPB principles and rights to their drone DII processes, and examines how these principles and rights could be reflected in concrete, drone-related internal policies adopted by these organisations.

**Supplementary Information:**

The online version contains supplementary material available at 10.1186/s41018-025-00174-z.

## Chapter 1: Introduction

In a bid to fulfil their noble call for humanitarian intervention, governments and humanitarian organisations have been and are increasingly reliant on data and technology to map and contain the effects of crises, make intervention decisions, manage logistics, respond to calls for help, and even anticipate the occurrence of natural disasters in specific locations (Beduschi 2022). Drones, also called Unmanned Aerial Vehicles (UAVs), constitute one of these new technologies collecting and generating important data to support humanitarian action, hence the term “humanitarian drones” (Sandvik and Lohne 2014). They are remotely controlled aircraft usually equipped with devices to capture and record visual and audio data for monitoring and mapping operations and are widely used in humanitarian action, especially for locating survivors, strategic mapping, emergency surveying, and other forms of disaster assessment. Sophisticated equipment like high-resolution cameras can be attached to a drone to generate clear, high-resolution aerial photographs (Lei et al 2017) from which individuals, groups of persons, or an entire community can be identified. In Malawi, the case study of this paper, drones are often used for disaster resilience, collecting aerial data which can be analysed to predict or calculate the impact of an eventual disaster, an example being the flood modelling objectives of the UNICEF Malawi drone corridor launched on 29th June 2017 in Kasungu, Malawi.^1^

As observed by Sandvik and Lohne (2013), technological innovations in crisis response may intersect with moral values, norms, and commitments and may challenge humanitarian principles. Solymosi et al. (2023), arguing from a geodata standpoint, also point out that the collection and dissemination of geographically explicit data of communities may expose them to unintended privacy violations, for instance, where the data is wrongly believed to have been appropriately anonymised. Because affected populations can be simultaneously harmed and helped using data, there is a need to ensure that humanitarians understand and mitigate risks related to their data processes. These include traditional risks involved in data collection and use, such as the disclosure of personally identifiable information (PII) and security challenges for those collecting and handling data in unstable contexts. Consequently, drones have been constantly associated with privacy and data protection concerns (Hovsha and Brian 2022; Wang et al 2021). As mentioned above, their on-board equipment may have the ability to capture, store, or relay sensitive information about people or communities. Also, their capacity to fly at great heights rendering them invisible from the ground makes them sophisticated tools for mass surveillance, spying, or other forms of image processing without consent or knowledge of the people on the ground. There is therefore an important need for privacy and data protection safeguards when deploying drones to human settlements for any form of intervention, which is the assertion on which this research was carried out in Northern Malawi. Two significant issues were encountered in this research, which make up the core subject of this paper.

First, previous research has established that in the face of their vulnerability and need for external aid, residents in disaster-risk zones in Northern Malawi do not care much about data protection and privacy. This was the main finding of a survey carried out in two disaster-risk districts of Northern Malawi to investigate their views and perspectives on drone technology in their communities for disaster preparedness and resilience (Alunge 2024). As a result, the residents cannot reasonably be expected to be very engaged in questions related to the protection of their data, nor in engaging their data protection rights, which places the burden of ensuring responsible and ethical use of their data entirely on the humanitarian organisations collecting and processing these data. And arguably the most comprehensive and available means of guaranteeing responsible drone data collection and processing in Malawi would be their subjection to the data protection principles and rights listed in the 2023 Malawi Data Protection Bill (MDPB).

However, this raises the second dilemma: drone data collected within these communities are mostly demographically identifiable information (DII), and as a result are not covered by the Bill which, like most contemporary hard law on data protection, expressly applies only to personally identifiable information (PII). With national data protection law being a robust, legally binding means of ensuring responsible data processing, the exclusion of DII from its scope leaves the latter without directly applicable and enforceable rules of data governance. Against this backdrop, the research paper seeks to examine the hypothesis that despite their substantive inapplicability to drone DII, the principles and rights of the MDPB remain relevant and could feasibly be applied to humanitarian drone processes in Malawi. To this end, the paper first sought to examine the feasibility of applying these principles and rights to practical humanitarian drone data processes. To achieve this, a series of twenty (20) semi-structured interviews with humanitarian and government officials were carried out to sample their perspectives regarding the practical and feasible application of the principles and rights in the MDPB to their drone processes. The results showed that the interviewees largely believed that the data protection principles and rights of the MDPB could indeed regulate their drone DII activities to ensure responsible data processing. This then prompts the proposal of some regulatory adjustments which, if adopted by Malawian lawmakers, would strongly induce the application of these MPDP principles and rights to humanitarian drone DII. The paper equally examines how some of these MDPB principles and rights could be reflected in internal drone data policies adopted by humanitarian actors in Malawi which would foster best practices as regards the responsible use of drone data.

The paper therefore adopts the following structure: following this introduction, Chapter 2 discusses DII in more detail, highlighting its significance in humanitarian action. Chapter 3 then briefly discusses the concept of data protection, presents an overview of the Malawi data protection framework (i.e. the MDPB), highlighting the challenges related to its inapplicability to DII. Chapter 4 then presents the semi-structured interviews with humanitarian and government officials and discusses the findings, while Chapter 5 proposes and discusses some regulatory changes which could strongly induce humanitarian organisations to apply the MDPB principles and rights to their humanitarian drone DII processes. Chapter 6 then proposes and discusses policies which these organisations can adopt with regard to their drone DII processes which would directly reflect some selected principles and rights of the MDPB, fostering responsible drone data collection and processing. Chapter 7 shall then present a general conclusion to the paper.

## Chapter 2: demographically identifiable information

To discuss demographically identifiable information (DII) especially with regard to its relationship with data protection law, it is important to first understand the concept of personally identifiable information (PII), also known as personal data (Schwartz and Solove 2014a, b). The MDPB, in its Article 2, defines personal data as ‘any data relating to an identifiable natural person which, directly or indirectly, by reference to an identifier such as a name, an identification number, location data, an online identifier or one or more factors specific to the physical, physiological, genetic, psychological, cultural, social or economic identity of that person.’ This is essentially the same definition adopted in other mainstream data protection and privacy regulations or policies, notably the 2016 European Union’s General Data Protection Regulation (GDPR)^2^ as well as the 2015 UNICEF Policy on Personal Data Protection.^3^

DII on the contrary has been defined as ‘either individual and/or aggregated data points that allow inferences to be drawn that enable the classification, identification, and/or tracking of both named and/or unnamed individuals, groups of individuals, and/or multiple groups of individuals according to ethnicity, economic class, religion, gender, age, health condition, location, occupation…’ (Raymond 2017). Also known as ‘group data’, DII can include PII, geographic and geospatial data, environmental data, survey data, and/or any other data set that can—either in isolation or in combination—enable the classification, identification, and/or tracking of a specific demographic categorisation constructed by those collecting, aggregating, and/or cross-corroborating the data (Raymond 2017). In other words, while PII would distinguish and enable the identification or recognition of an individual from within a group of persons, DII would tend to distinguish a community or group of persons from within a (larger) group of persons or communities. It would also include data like high-resolution images of a community depicting housing structures, monuments, shrines, agricultural and agropastoral patterns, religious practices, dress codes of the people which (when combined with other available datasets) can enable the identification or recognition of that community from other communities (Alunge, 2024). An example will be images showing people dressed identically with sparsely spaced buildings, from which one could infer the presence of school grounds. As drones are (usually) equipped with cameras, sensors, and other sophisticated measuring capabilities, they are very likely to capture such DII when deployed to communities for humanitarian purposes.

It is important to mention at this stage that the concept of a ‘group’ has been subject to a significant amount of scientific debate. When faced with the question of what constitutes a group, authors offer different perspectives as to the kinds of groupings, ranging from traditional notions of social or political collectives and ethnic groups to people who face a common threat [e.g. refugees] or share a common interest (Taylor et al. 2017). Other authors talk of ‘informational’ groups created by algorithms which aggregate datasets harvested from vast data sources (Reviglio and Alunge 2020). Notwithstanding the importance and relevance of these new notions of a group—and the privacy or data protection challenges they raise—this paper examines the group as a community with a relatively defined set of people as its residents, who share a similar culture or livelihood, and above all are faced with the same threat: natural disaster. Given its focus on humanitarian drones, and leaving aside other DII created by grouping together individual digital profiles using big data analytics, the DII discussed in this paper will for a large extent be drone-collected data about or related to the characteristics of human settlements. Taking cue from the definition of ‘group privacy’ advanced by Floridi (2017) as a ‘right that is held by a group as a group rather than by its members severally…it is the group, not its members, that is correctly identified as the right-holder…’ this paper discusses DII as data relating to the community as one entity, the processing of which can affect a large part of if not the entire community.

### Significance of DII in humanitarian action

Misuse of DII could have a direct and harmful impact on a specific group or community of people. An example is the incident concerning Harvard’s Signal Program on Human Security and Technology, which ran a project analysing satellite data to identify settlements which had been most affected during the 2011 crisis in Sudan. The researchers later discovered that their work was being hacked and used by hostile entities on the ground to better target their enemies (Taylor 2017). Also, supply chain information of supplies to a refugee campsite can determine the presence of lactating mothers, while important for providing maternity care, would also be a vital tool in the hands of malicious persons harbouring ambitions to limit or prevent childbirth by refugees in a country they seek refuge in (Raftree 2019). The above shows situations warranting the observation of data protection safeguards during DII collection, processing and sharing; which should equally be the case with humanitarian drone data in Malawi. The following chapter discusses the concept of data protection and presents the Malawi data protection framework together with an overview of its principles of data processing and rights.

## Chapter 3. data protection law and the Malawi data protection framework

The origins of data protection law as it is known today can be traced back to the privacy laws of northern European countries enacted in the 1970 s, the Council of Europe’s Convention for the Protection of Individuals with Regard to Automatic Processing of Personal Data of 1981, and the Fair Information Practice Principles (FIPPs) conceived in a 1973 Federal Government report by the US Ministry of Health, Education and Welfare. These developments were prompted by the realisation that the traditional right to privacy was seriously challenged in the face of modern technologies of large-scale, automated data processing (Van der Sloot 2017). These instruments originally advanced sets of principles and rights to protect privacy within computational data processing, but over the years have developed the ability to protect all other individual rights from harm which may arise from collecting and/or processing (personal) information (De Hert and Gutwirth 2009). Today, these principles form the core of several key data protection regulations or policies around the globe^4^ and are equally inscribed in the MDPB, adopted by the national parliament on 7th December 2023 and which is poised to be, once it is assented into law, the main piece of hard legislation governing PII processing in the country. Table 1 presents an overview of the data processing principles and rights provided for in the MDPB.

**Table 1:** Data protection principles and rights in the MDPB

Principles of data protection	Rights available to individuals
Fairness and Legitimacy (Article 8): Personal Data should be processed fairly and lawfully. The lawfulness of the processing requires a legal basis for processing operations to take place.	Right to be informed (Article 15). For purposes of transparency, the individual should be provided with relevant information when their data is collected, which includes the identity of the organisation, why the data is collected, circumstances in which the data may not be processed confidentially, how long the data will be stored, who it will be shared with, as well as any rights they may have on the data.
Proportionality (Article 30(3)(d)): The amount and type of personal data collected and processed, or measures used for collection and processing, should be appropriate for the pursued aim.	Right of Access (Article 19). Individuals should be able to make an access request, orally or in writing, to the organization, by which the organization shall present their personal data to them for any review or verification.
Defined purpose or purpose limitation (Article 9): Personal data should be collected and processed for a specific and legitimate purpose and should not be processed in a manner incompatible with that purpose (purpose limitation);	Right of correction/rectification (Article 21): Following access and reviewing of their data, the individual may request that the organisation rectifies any inaccuracies in the personal data.
Data minimisation (Article 10): Personal data collected and processed should be adequate, relevant and limited to what is necessary for the that purpose. In other words, data which is not needed should not be collected.	Right of erasure/deletion (Article 22): Following access, the individual may, if they have a valid reason, request that the organisation deletes the data from its files
Data quality (Article 11): Personal data should be accurate and, where necessary, kept up-to-date as needed for the purpose for which it was collected.	Right to object to processing (Article 24): Similar to the right to erasure, the individual may, if they have a valid reason, request that the entire or part of the data processing process be halted.
Retention or Storage limitation (Article 12): Personal data should not be stored for a period longer than is necessary to achieve the purpose for which the data was collected.	Right to complain against data protection violations (Article 44): Individuals also have the right to bring a claim in court or with a data protection authority or to any authority designated to oversee the application of the country’s data protection law.
Data security (Article 9): Organisations collecting personal data should have appropriate technical or organisational security measures in place to guarantee security (Data integrity and confidentiality).	

It should be noted however that data protection rights are generally not absolute and have to be balanced with other rights and public interests (Galetta and De Hert 2017), and Article 26 of the MDPB accordingly invalidates the above rights in certain special instances. For example, these rights may not be invoked by an individual if their PII data is being collected or processed in the interest of national security, investigation or prosecution of a criminal offence, pursuing a national economic or financial interest, public health, social security, or judicial proceedings. While disaster resilience is not expressly stated as such a special instance in the MDPB, it could conveniently be matched with a national interest, and on these grounds, the data protection rights of affected residents may be waived if guaranteeing them during a specific humanitarian process proves logistically unfeasible or impossible. In fact, as pointed out by Zwitter and Gstrein (2020), data protection and privacy are human rights that can be derogated from during crises, or can be temporarily reduced when a public emergency calls for it.

### The challenge: incompatibility of the MDPB to (drone) DII

While the above principles and rights in the MDPB constitute a formidable arsenal to ensure responsible and accountable data processes, they face significant challenges with regard to DII (including drone data) in that they are substantially limited in their scope to apply only to PII**.** Current data protection frameworks are designed to apply only to identified individuals as right holders (Purtova 2017); prominent data protection regulations all expressly limit their scope to PII,^5^ while some frameworks expressly exclude DII from their scope of application. For example, Paragraph 8 of the UNICEF Policy on Personal Data Protection states: ‘The following topics are outside the scope of this Policy: … (b) data that can identify a group, demographic or community, but *not* an individual’. In line with this trend, Article 3 of the MDPB states that the Bill applies to ‘the processing of personal data in Malawi…’. Considering its definition of personal data in Article 2 discussed in Chapter 1, it appears clear that the MDPB applies to the processing of PII to the exclusion of DII. As the latter includes drone data through which individuals cannot be identified, it logically follows that such data is not covered by the Bill.

A likely reason for this exclusion is the difficulty linked with conceptualising the interest of a group as one interest, while the group is a collective of individuals with usually diverse interests or interest levels. For example, what happens if one or more members of the group consent to a specific DII processed and other members of the same group do not? As a matter of fact, the dynamics by which a group can enjoy privacy is still debated among scholars. Bloustein and Pallone (2018) point out that for a long time in privacy literature, the group had been conceptualised only as a collection of individuals with individual privacy interests. The only fixed entity is therefore the individual, with group privacy being nothing more than the sum of ‘individual privacies of each member of the group. Floridi (2017) in contrast, argues that groups as a whole can have a right to specific kinds of privileges [not excluding privacy] regardless of whether or not each member of the group has the right to that kind of privilege, an example being the right to self-determination which can be available to a population but not to each individual within that population.

As pointed out in the introduction, this inapplicability of the MDPB principles and rights to humanitarian drone DII leaves the latter without a comprehensive, legally applicable data processing framework in the country. This is particularly concerning as protection within humanitarian contexts is expected to be at the highest possible standards considering the vulnerability and fragility of affected persons and the sensitive nature of the data collected and processed (Kuner et al. 2020). It is in response to this assertion that this paper seeks to establish the practical relevance of the MDPB principles and rights to humanitarian drone data processes and investigate strategies through which humanitarian organisations in Malawi could be strongly nudged or induced into applying them to their drone DII processes. First, the paper seeks to establish whether these principles and rights could feasibly be applied by humanitarian organisations collecting and processing DII in Malawi. To attain this objective, the researcher interviewed a selection of humanitarian and government officials who collect and work with drone data in Malawi to sample their perspectives on the applicability of the MNDP principles and rights to their drone activities. The organisation and findings of these interviews are presented in the following section.

## Interviews with humanitarian professionals and government officials in Malawi

### Interview structure and participants

The aim here was to establish the extent to which humanitarian professionals and government officials working with drone data in Malawi believed (or not) the MDPB principles and rights could feasibly be applied to their drone DII processes, especially with regard to logistical challenges. To this end, the study used semi-structured recorded interviews: the participants were presented with a questionnaire containing several statements on the applicability of the principles and rights provided in the MDPB to their drone data collection and processing practices. They were invited to select how much they agree or disagree with the statements, from extreme positive to extreme negative (i.e. whether they Strongly Agree, Agree, are Neutral, Disagree or Strongly Disagree) and prompted to discuss their choice further.

A total of twenty (20) officials working with humanitarian organisations and Malawi government departments were selected for the interviews, based on their direct involvement in the collection, processing, use of or making decisions based on drone data in disaster-affected or disaster-risk locations in Malawi. These included drone operators, policy makers and administrators in active service or who have worked on drone operations. Due to availability issues, 8 interviews took place online via Microsoft Teams, while the other 12 took place in person. The humanitarian officials interviewed worked at the time with UNICEF, the World Food Programme (WFP) and the United Nations Country Office of Malawi, while the government officials were from the Department of Disaster Management of Malawi (DODMA) and the Malawi National Water Resources Authority. It should be highlighted here that the selection of humanitarian professionals alongside government officials was only intended to have, within our dataset, opinions which were inclusive of most bodies working with drone data collected and processed within a humanitarian context in northern Malawi. There was no intention to highlight any differences or similarities between the perspectives of government officials and those of the humanitarian professionals.

Table 2 shows some of the questions presented to the participants. (For the complete questionnaire and answer percentages, please see Annex 1).

**Table 2:** Main questions in the questionnaire presented to the interview participants

On categories of data collected and processed	- Can UAVs deployed to communities for flood or disaster resilience collect data which is personally identifiable information (PII)? Or can they collect data which is demographically identifiable Information (DII)?
Sensitive information	- Do you believe (some) data collected by UAVs of a community could be information which the community residents consider sensitive?
Information derived from drone images	- Do you think high-resolution aerial images or other related data taken via UAVs of a community can tell accurate information about the residents like their main religion, ethnicity, principal occupation, food security levels, economic situation?
Data accuracy	- Could UAVs data lead to produce unfair or biased interpretations and influence decisions which could be detrimental to the community?
Data minimisation	- Can humanitarian UAVs collect extra data of that community than was originally needed or intended?
Purpose limitation	- Do you believe high-resolution aerial images of people or their community, or similar aerial data collected through UAVs, could possibly be used by government or the collector organisation for another, unintended purpose than the original purpose for which the UAVs were deployed?
Storage limitation	- Do you think there should be a time limit for the collector organisation to store high-resolution aerial images or similar data of a community and/or its residents, after which the images should be deleted?
Data security	Should the organisation have a responsibility to audit the technology of the UAVs supplied to them by donors to ensure it is data protection compliant (e.g. the donors do not have discreet access to the data)?
Rights of the community (as a group)	- Should community residents always be informed about the details of the UAV data before the UAVs are flown (right to be informed)? Should their informed consent be sought before flying the drones? Should they be able to ask the collector organisation to show or present to them all the aerial images or other data it has about them and/or their community (right of access)? Should they be able to request that the data be deleted (right to erasure)?

### Key findings/results and discussion

Table 3 represents a summary of the key findings from the interview discussions guided by the questions in Table 1 above.

**Table 3:** Summary of key findings

Data protection principles	Participants who agreed—with supporting reasons	Participants who disagree—with supporting reasons	Participants who remained neutral
Data quality	14 - Drone data processing may lead to biased or unfair results e.g., wrongfully classifying plants lying horizontally as dead plants	3 – Organizations’ image processing systems or algorithms are always properly trained.	3
Data minimisation	20 - Drones unavoidably tend to collect extra data than intended as they fly over assigned surface areas.	0	0
Purpose limitation	18 - Drone data originally collected for disaster preparedness could be reused for developmental purposes, as this is compatible with original purpose of collection	2	0
Storage limitation	3	14 - Drone data is not as sensitive as PII, and its collection is expensive and logistically challenging	3
Data security	20 - Organisations should have a responsibility to audit drones supplied to them by third parties before deploying them to the field.	0	0
Right to be informed	20 - Organisation should always inform the residents before flying their drones, community awareness guarantees the safety of the data collectors in the field	0	0
Right to informed consent	18 - Consent should always be sought from the community leaders prior to flying drones	2	0
Right of access	15 – A community, through its leaders, can request an organisation to grant them access to drone data collected about their community.	3	2
Right to erasure	7	11 - community leaders should not have the power to request the deletion of drone data collected from their community by (humanitarian) organisations.	2
Right against automated-decision making	19 – A human should always be involved at some point in the collection, processing or decision-taking phase of the data process.	1	0

From the discussions guided by the above questions, the following conclusions were drawn. First, all participants agreed that humanitarian drones, during operational response, do collect (and sometimes process) DII. All agreed that drones could collect more data than originally intended, which highlights a data minimisation challenge. Also, out of the 20 participants, 18 agreed (10 of whom strongly so) that aerial data collected could possibly be used for different purposes than for which the drones were originally deployed where these new purposes were still oriented towards disaster resilience or development (purpose specification/limitation challenge). However, only 6 participants felt that their organisations should have a time limit to store drone data, while 14 were against a storage time limit generally because, Malawi being a relatively peaceful country, they felt aerial drone data of a community is generally not sensitive as opposed to PII. Also, because drone data collection is usually an expensive and logistically challenging process. As regards security, all participants agreed that their organisations should be responsible for auditing the drones they acquire from private third parties for security leaks before deploying them to the field, while 19 participants out of the 20 were in favour of carrying out a data protection impact assessment to identify any data-related risks and plan on their mitigation before deploying the drones for operations, without prejudice to any established exceptions in the event of an emergency. All participants were in favour of the presence of a person in charge of data protection compliance among their staff to advise on privacy and other data protection concerns regarding drones.

With regard to community rights, all 20 participants agreed, with 16 of them strongly so, that the residents should always be informed before drones are flown over their community (right to be informed). Here, the participants stressed the point that not only is this ethically and culturally appropriate, but such community awareness also ensures the safety of the data collectors who, as confirmed by the participants, run a real risk of physical assault from the residents who may find the drone flight suspicious, e.g. as an instrument secretly taking measurements of their farmlands or otherwise spying on them. In a similar manner, 18 of the 20 participants believed the consent of the community (through the community leaders) must also be sought before flying the drones (right to provide consent). Further, 15 of the participants agreed, as opposed to 3 dissenting and 2 remaining neutral, that residents, though through the authority of a chief or local leader, should be able to have access to drone data imagery which organisations collected from their community (right of access). However, only 7 of them agreed, as opposed to 11 dissenting and 2 remaining neutral, that the residents should have the power to request the deletion of these data (right to erasure). The dissenting 11 in this case stated their main motivation being high costs, administrative and technical challenges deploying data collectors to disaster-affected areas, and the collected data can always be useful for future disaster-related assessments. Further, 19 of the 20 participants were against purely automated decisions taken on drone imagery without the involvement of a human at some point in the data cycle process (right against automated decision-making). And 14 agreed to the possibility that drone data processing, especially when done using algorithms, may produce biased results.

The above results show that the participants, for the most part, agreed that the above-stated principles of data processing and rights available to individuals incorporated in the MDPB could regulate the drone data collection and processing operations of their organisations. While the levels of acceptance of these principles and rights varied among the participants, there was general agreement that they were important and feasible considerations for humanitarians in terms of ensuring ethical and responsible drone data processing. It is however important to acknowledge here that due to the relatively small number of participants successfully interviewed, this paper does not intend to portray these findings as definitive or as a thoroughly researched, objective presentation of the status quo on this topic. Nevertheless, it certainly highlights a trend of thought among humanitarians, at least those working in Malawi, on the applicability of hard data protection law on drone data and other DII, which lays the groundwork for further discussions and research on the topic.

The above discussion leads to the conclusion that the MDPB principles and rights can acceptably regulate the processing of humanitarian drone DII in Malawi. However, with the MDPB limited in its scope to directly apply only to PII, the question arises as to how humanitarian organisations in the country could be compelled or nudged into incorporating the said principles and rights into their drone DII processes. The following Chapter attempts to address this question by proposing and discussing some recommendations in the form of regulatory actions (by the Malawian legislators) and policies (adopted by humanitarian organisations) which could strongly induce the application of the MDPB rights and principles to humanitarian drone DII processed in the country.

## Regulatory actions to nudge the application of the MDPB on of humanitarian drone DII

### Involving the Ombudsman

A regulatory action which could potently nudge humanitarian organisations towards applying the MDPB principles and rights to their drone data collection and processing activities in Malawi would be the express involvement, by a legislative or other relevant Act, of the national Ombudsman as an alternative complaint mechanism beside the Data Protection Authority.^6^ The Office of the Ombudsman in Malawi was created by the Constitution of 18th May 1994 with the mission to ‘…investigate any and all cases where it is alleged that a person has suffered injustice and it does not appear that there is any remedy reasonably available by way of proceedings in a court or by way of appeal from a court or where there is no other practicable remedy’ (Article 123(1)). Its functions and duties are laid down in the Ombudsman Act of 31 st December 2014. In practice, the main role of the Ombudsman’s Office, as is the case in other countries with a similar authority, is to investigate and report on injustices by public, para-public or politically influential and powerful corporations in a country against whom litigation may be too expensive, burdensome for the average citizen, or is evident of a power imbalance. After investigations and in the event of an established injustice on the part of the corporation, the Ombudsman may try to negotiate a compromise between the complainant and the corporation, or report the case to the Attorney General or Director of Public Prosecution (Article 8(1), Malawi Ombudsman Act 2014). It also publishes yearly reports on its investigations, available on the Office’s website^7^: Though, unlike the Data Protection Authority, its decisions do not have the binding force of law i.e. it cannot order sanctions against an organisation, injustices established and reported by the Ombudsman are usually very influential on public opinion and can have a significant impact on an organisation’s credibility.

This paper hereby suggests that the involvement of the Ombudsman in humanitarian drone data processes can bring significant dynamism in applying the principles and rights in the MDPB to humanitarian drone data processes. This is principally due to three reasons: first, the Ombudsman enjoys a wider investigative jurisdiction as opposed to the Data Protection Authority. The latter’s entire competence is strictly limited to the subject matter of the Bill, i.e. processing of PII, and it therefore cannot receive complaints out of this scope. Meanwhile, the Ombudsman, as per the Constitution, is competent to investigate *any* injustice, which means it is equally competent to investigate complaints about DII processing, including drone data. Secondly, the MDPB appears to operate under the legal principle of privity in tort regarding access to remedies, which generally states that the only person who can enforce a right is the right-holder (Stevens 2007). Unlike other data protection jurisdictions,^8^ the Bill does not provide for third-party representations, which means the Malawi Data Protection Authority cannot receive a complaint against a data processing organisation from a third party who is not affected by the organisation’s processing actions. Only an affected party or their legal representative can lodge a complaint. This limitation is conveniently surmounted by the Ombudsman, who can receive and investigate even anonymous complaints against an organisation. Hence, public interest activists or even anonymous persons may lodge complaints to the Ombudsman regarding unsatisfactory drone data or other DII processing by humanitarian organisations in Malawi. And finally, the negative publicity of featuring in an Ombudsman investigative report would serve as an incentive for data processing organisations not only to comply with national law but also to adopt ethical, human-centric data practices, like the principles and rights in the MDPB.

### Nationally declaring humanitarian data as highly sensitive data

Another regulatory action which could probe humanitarian organisations into applying MDPB rules on their drone data processes in Malawi could be a national declaration, by decree or other form of secondary legislation, qualifying humanitarian data in the territory as highly sensitive data which can only be collected and processed under strict obligations of care. In the face of such a development, humanitarian organisations in the country would be compelled to apply the highest, most robust protective measures for the data they collect, with the available framework offering the most comprehensive of these measures in the country being the MDPB.

This proposal is inspired by the current trend in most contemporary data protection law frameworks which expressly categories specific data as ‘sensitive (personal) data’ which, due to their delicate nature, can expose individuals to higher risks of personal harm if these data are compromised^9^ (De Hert et al 2009). There is therefore an expectation for data processing organisations to demonstrate stricter standards of care when processing data of this category. And considering their manifested reception of the MDPB principles and rights to oversee their drone data processes, as shown in the previous section, these stricter standards of care would certainly probe humanitarian organisations to ultimately incorporate into their drone processes the data processing principles and rights in the MDPB as the current, most comprehensive legislation and highest standard of (digital) data governance in the country.

After discussing regulatory actions which could nudge humanitarian actors into applying MNDP principles and rights to their drone DII processes, the paper now turns to examine how these principles and rights could be reflected in drone-related strategies adopted by these organisations. The following Chapter hence proposes some policies directly reflecting some MDPB principles and rights which humanitarian organisations could internally adopt to foster responsible drone data collection and processing in Malawi.

## MDPB principles and rights reflected into humanitarian drone DII policies

### Data minimisation (Article 10 MDPB): establishing no-fly zones over sensitive areas in local communities

Concepts of what information should be considered sensitive or private may vary between cultures, age groups, interest groups and other demographics (UN-GGIM 2015; Gevaert 2018). Local idiosyncrasies might vary amongst communities depending on history, political setting, or cultural practices which can influence attitudes and behaviours, and in turn introduce special privacy concerns unique to local contexts (Kim and Kwan 2021). Therefore, the ethical cultural and local contexts should also be considered when collecting and processing data (Georgiadou et al 2019). In Sub-Saharan Africa, for example, traditional shrines and cults are usually sacred and are accessible only to certain dignified members of a community, which implies data relating to them could be considered sensitive by the community residents. As such, members of the community may not want details of what happens within these sacred settings, sometimes even their location, to be known outside of the community. They therefore may react negatively to the collection of aerial, high-resolution images by external entities depicting these settings (Alunge 2024). In the case of Malawi, some participants in the interviews presented in Chapter 4 confirmed that they have worked in areas where graveyards and local shrines were considered sensitive by the local residents.

In this light, applying Article 10 of the MDPB would translate into humanitarian organisations taking measures to ensure that during aerial terrain surveys, their drones are prevented from filming areas which the community considers sensitive. Technical measures could be put in place to ensure the geographical coordinates of these areas are registered within the drones as ‘no-fly zones’ and equipped with trigger warning systems which alert a drone pilot when the drone in flight goes close to these areas. This will further enhance data minimisation within humanitarian drone data processes. A potential setback of this policy, however, may be that blocking these areas could inadvertently increase the risk of them being prime targets in the event of a malicious attack on the drone database which would reveal, to the attacker, how sensitive they are to the community. This risk could be mitigated by not making public the reasons for this blocking, with access to such information strictly limited to internal circulation; or if it is shared externally, this should be done only upon a formal application of an information request by the third party, who may be subject to some screening before the information is given.

### Data quality (Article 11 MDPB): ground-truthing and involving human action to limit bias

**Fig. 1:**
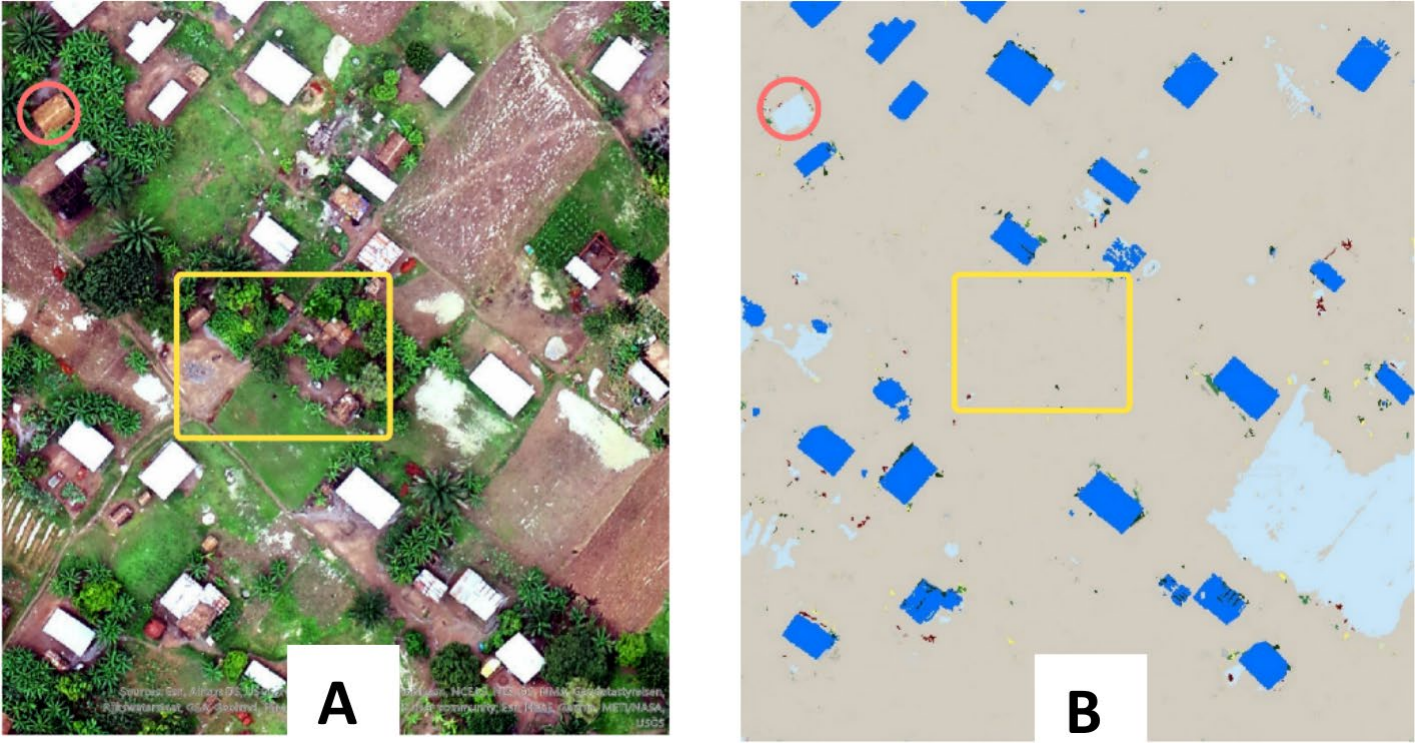
Image of how biases emerge from UAV classification of buildings by their roof types using AI. **A** Drone-captured image of an area of interest with buildings having two types of roofing materials. **B** A classification outcome of **A** using AI where buildings with one of the types of roofing materials are either misclassified (circled in red) as a non-buildings or not identified at all (several buildings in the area in yellow) while buildings with the other roofing material are identified consistently (in blue). Decisions based on housing demographics as viewed on the AI-generated image would not have accurate data on the number of households in the area; hence, a bias towards those living in those houses. Source: Masinde et al. (2023)

As illustrated in the interview results presented in Chapter 4, 14 of the participants believed processing drone-acquired data with the aid of modern technologies, while cost and time-efficient, could generate inaccurate or biased data on which decisions are taken which impact the target aid receivers. This has been a major concern in humanitarian action with the increased employment of artificial intelligence (AI) by organisations to assist aid processes. With AI systems, machines are trained to make sense of data through machine learning (Flach 2012) to enrich knowledge bases which are accessed via computer softwares and used to solve problems usually requiring specialised human expertise. While progressively transforming humanitarian action, AI also raises concerns of data quality and, ultimately, bias. If the data used to train AI systems is incomplete or contains errors, the outcomes (e.g. determination and classification of vulnerability indicators) might be equally poor in quality (Beduschi 2022). Drone-captured images can and are equally used to train AI systems, and due care should be taken to ensure the quality of the data generated from these images which are then fed into these systems. Figure 1 below illustrates an example of AI data (image B) generated from a drone-captured image (image A) which fails to identify, as houses, structures with lower-quality rooftops. In this instance, aid decisions based on households in the specific area will likely not consider the people living in those houses.

To safeguard data quality and reduce risks of bias in accordance with Article 11 MDPB, humanitarian organisations in Malawi could adopt policies against any image processing without involving human action to check the quality of the AI-generated image. Further, during the data collection phase, they may also consider ground-truthing, which is the establishment of the accuracy of remotely sensed data by field visit (Black et al 2008). However, ground-truthing is usually expensive and time-consuming, requiring visits to as many sites as possible to have sufficiently large training datasets, and raises logistical challenges like accessing remote locations, communication problems, equipment failure, or physical stress (Kirui et al 2021).

### Individual data protection rights (Articles 15–24 MDPB): implementing a community rights mechanism for drone data processing

As illustrated by the Chapter 4 interviews, humanitarian actors in Malawi largely accept the applicability of the data subject rights listed in the MDPB on their drone data processes in the country. However, during the interview discussions, they all admitted that their organisations did not have systems or mechanisms in place to act on requests by residents to enforce rights. To guarantee the rights of individuals under the MDPB, humanitarian organisations operating in the country could have in place mechanisms and policies to manage related rights of the community over drone-collected and processed data relating to their community. However, as discussed in Chapter 3, the legal conception of privacy and data protection revolves around individuals as rightsholders, with the concept of a legally founded and enforceable group or community right to data protection not yet existent, though subject to significant philosophical debates (Bloustein and Pallone 2018; Floridi 2017). This is understandable because a group is made up of individuals with diverse interests, and it is not clear how to proceed if one individual in the group may wish, for example, to grant consent for processing a group data set, while another individual of the group may not.

Within a humanitarian context, one way to overcome this challenge could be to work with community leaders (e.g. chiefs, quarter-heads, etc.) as representatives of the community who can act on behalf of the community. For example, to guarantee the right to be informed of a data collection or processing activity (Article 15 MDPB), the organisations may present details about the drone flights (show sample drone-captured pictures, their objective, who it may be shared with, how it will be stored, etc.) to community leaders during pre-flight awareness campaigns. These community leaders can be consulted to provide consent for drone data collection and processing on behalf of the community; Kuner et al (2020) advance the idea of ‘consent of the community’ or ‘consent of authorities’ as a plausible alternative to individual consent offered by contemporary data protection regimes. This would also be easier for the organisation, as it will be unfeasible to request the consent of every individual in the community. With community leaders identified as the rightsholders’ representatives, it would be easier for humanitarian organisations to work with them regarding community data rights. However, organisations should, amid possibilities of power imbalance between community leaders and residents, political unrest or conflicts of authority within the community, always carry out due diligence investigations to ensure that these representatives at least actually represent the majority of the community.

As for the right of access (Article 19 MDPB) and/or deletion (Article 22 MDPB), the organisations may put in place technical mechanisms to receive, examine for authenticity and, if feasible or reasonable, execute such requests by community leaders. This could also be facilitated by the presence of a Data Protection Officer or someone in charge of data protection or privacy, a similar position which, as revealed in the above interview results, was present in the field office of only one of the participating organisations.

## Chapter 7: Conclusion

Drones constitute a key component of new technologies used to disaster resilience processes, providing humanitarian actors with cheaper and efficient means of collecting and processing aerial data of target communities. However, they raise several legal and socio-cultural challenges, among which, significantly, are challenges related to data protection. Many countries have functional data protection laws to regulate data processes, these inopportunely apply only to PII. This situation is currently reflected in Malawi, where several humanitarian organisations collect and process drone data for disaster resilience in the country, while the country enacted the MDPB but which is limited in scope to PII. As a result, aerial drone images of a community on which individuals cannot be identified, which would be DII and hence non-PII, will generally fall out of the scope of the MDPB. Meaning the data protection principles and rights provided in the MDPB cannot be legally applied to humanitarian organisations processing drone DII, leaving the latter bare of a comprehensive, binding regulatory framework in the country. This is a disturbing situation considering that DII processes in humanitarian contexts, just like PII, could equally present significant risks of harm to target communities.

It is within this context that this paper sets out with two objectives: first, to push the limits of the status quo and make a case for the feasible applicability of the principles and rights provided in the MDPB on humanitarian drone DII processing in Malawi. This was attained by interviewing 20 humanitarian professionals and government officials working with drone data in the country to gather their perspective on the practical applicability of the MDPB principles and rights on their drone data processes, to which the responses were largely in the affirmative. Following this conclusion, the paper then moved to its second objective: it proposed some regulatory modifications which the Malawian legislators could adopt to nudge humanitarian actors towards applying the MDPB principles and rights to their drone DII processes in the country, before examining how these could translate into concrete, drone data-related policies which can be adopted by the humanitarian actors within their organisations.

## Supplementary Information

Supplementary Material 1.

## Data Availability

The datasets generated and/or analysed during the current study are available in the Dutch national centre of expertise and repository for research data (DANS) at 10.17026/SS/HU8ZNS on reasonable request.

## Abbreviations

DII: Demographically identifiable information
GDPR: General Data Protection Regulation
MDPB: Malawi Data Protection Bill
UAV: Unmanned Aerial Vehicle
PII: Personally identifiable information
UNICEF: United Nations Children’s Fund

## Footnotes

1 In June 2017, the Government of Malawi and UNICEF launched a 40-km-radius air corridor at the Kasungu Aerodrome in Central Malawi to as a base to test drones for various humanitarian purposes including flood modelling. Aerial images would be captured and run through AI image recognition and flood modelling software to predict flood impact and identify areas which will be most affected, aiming to better inform emergency preparedness through early warning. Retrieved from https://www.arm.com/blogs/blueprint/arm-unicef-malawi. Accessed 26/03/2023.

2 Regulation (EU) 2016/679 of the European Parliament and of the Council of 27 April 2016 on the protection of natural persons with regard to the processing of personal data and on the free movement of such data, and repealing Directive 95/46/EC (General Data Protection Regulation)—the GDPR.

3 Annex 1 Paragraph 11, UNICEF POLICY ON PERSONAL DATA PROTECTION, Document Number: POLICY/DFAM/2020/001. Effective Date: 15 July 2020.

4 These principles feature for example in the GDPR (Article 5), the Canada Privacy Act 1985 (Articles 7 and 8), the African Union Convention on Cyber Security and Personal Data Protection of June 27, 2014 (Article 13).

5 For example, Article 1.1 of the GDPR states that the regulation applies to the processing of personal data. Similar to Article 9.1(a) of the AU Convention on Cybersecurity and Data Protection.

6 The Data Protection Authority is the national, independent office in charge of overseeing the implementation of data protection rules laid down in the Malawi Data Protection Bill. Article 4 of the Bill designates the Malawi Communication Regulatory Authority as Data Protection Authority.

7 https://www.ombudsmanmalawi.org/documentation/investigations-reports Accessed 6th February 2024.

8 The 2018 General Data Protection Regulation of the European Union for example provides for the possibility of third party representations by non-governmental or public interest organisations to lodge complaints to the Data Protection Authority on behalf of affected individuals, even without the latters’ mandate.

9 See for example Article 9 of the EU GDPR which lists these as “special categories” of personal data.

## References

[CR1] Alunge R (2024) Assessing data protection perspectives among the residents of Rumphi and Karonga in Northern Malawi regarding the use of unmanned aerial vehicles (drones) for humanitarian intervention. International Conference on Innovations and Interdisciplinary Solutions for Underserved Areas (pp. 313–336). Cham: Springer Nature Switzerland

[CR2] Beduschi A (2022) Harnessing the potential of artificial intelligence for humanitarian action: opportunities and risks. International Review of the Red Cross 104(919):1149–1169

[CR3] Black K, Gallagher G, O’Brien P, Redmond J, Barrett F, Twomey M (2008) Dispelling myths: the true extent of recent peatland afforestation in Ireland. Coford Connects Environment No. 8, Dublin, Ireland. Retrieved from https://www.coford.ie/media/coford/content/publications/projectreports/cofordconnects/ccn08-env08.pdf. Accessed 19 June 2025

[CR4] Bloustein EJ, Pallone NJ (2018) Individual and group privacy. Routledge

[CR5] De Hert P, Gutwirth S (2009) Data protection in the case law of Strasbourg and Luxemburg: constitutionalisation in action. Reinventing data protection? Springer, Netherlands, Dordrecht, pp 3–44

[CR6] De Hert P, Gutwirth S, Moscibroda A, Wright D, González Fuster G (2009) Legal safeguards for privacy and data protection in ambient intelligence. Pers Ubiquit Comput 13:435–444

[CR7] Flach P (2012) Machine learning: the art and science of algorithms that make sense of data. Cambridge University Press

[CR8] Floridi L (2017) Group privacy: A defence and an interpretation. In: Taylor L, Floridi L, van der Sloot B (eds) Group privacy: new challenges of data technologies. p 83–100

[CR9] Galetta A, De Hert P (2017) A European perspective on data protection and the right of access.The unaccountable state of surveillance: Exercising access rights in Europe 21–43

[CR10] Georgiadou Y, de By RA, Kounadi O (2019) Location privacy in the wake of the GDPR. ISPRS Int J Geo Inf 8(3):157

[CR11] Gevaert CM (2018) Unmanned aerial vehicle mapping for settlement upgrading. PhD Thesis, University of Twente

[CR12] Hovsha J, Brian DO (2022) Drone use cases and their privacy impacts: a taxonomy. Tilburg Law Review 27(1):60–80

[CR13] Kim J, Kwan MP (2021) An examination of people’s privacy concerns, perceptions of social benefits, and acceptance of COVID-19 mitigation measures that harness location information: a comparative study of the US and South Korea. ISPRS Int J Geo Inf 10(1):25

[CR14] Kirui OK, Mirzabaev A, von Braun J (2021) Assessment of land degradation ‘on the ground’and from ‘above.’ SN Applied Sciences 3:1–13

[CR15] Kuner C, Marelli M, Barboza JZ, Jasmontaite L (2020) Handbook on data protection in humanitarian action. International Committee of the Red Cross

[CR16] Lei T, Zhang Y, Wang X, Li L, Pang Z, Zhang X, Kan G (2017) The application of unmanned aerial vehicle remote sensing for monitoring secondary geological disasters after earthquakes. In Ninth International Conference on Digital Image Processing (ICDIP 2017) Vol. 10420. SPIE, California, pp. 736–742

[CR17] Malawi Ombudsman Act (2024) Chapter 3:07, Commenced on 12 July 1996. Retrieved from https://malawilii.org/akn/mw/act/1996/17/eng@2014-12-31. Accessed 19 June 2025

[CR18] Masinde BK, Gevaert CM, Nagenborg MH, Zevenbergen JA (2023) Group-privacy threats for geodata in the humanitarian context. ISPRS Int J Geo Inf 12(10):393

[CR19] Purtova N (2017) Do property rights in personal data make sense after the Big Data turn? Individual control and transparency. J Law Econ Regul 10(2):64–78

[CR20] Raftree L (2019) A discussion on WFP-Palantir and the ethics of humanitarian data&nbsp;sharing. https://lindaraftree.com/2019/03/02/a-discussion-on-wfp-palantir-and-the-ethics-of-humanitarian-data-sharing/Accessed 10/02/2024

[CR21] Raymond NA (2017) Beyond “do no harm” and individual consent: reckoning with the emerging ethical challenges of civil society’s use of data. Group privacy: new challenges of data technologies, Springer, Champ, 67–82.

[CR22] Reviglio U, Alunge R (2020) “I am datafied because we are datafied”: an Ubuntu perspective on (relational) privacy. Philosophy & Technology 33(4):595–612

[CR23] Sandvik KB, Lohne K (2013) The promise and perils of ‘disaster drones. Humanitarian Exchange Magazine 58:28–30

[CR24] Sandvik KB, Lohne K (2014) The rise of the humanitarian drone: giving content to an emerging concept. Millennium 43(1):145–164

[CR25] Schwartz PM, Solove DJ (2014a) Defining ‘personal data’ in the European Union and US. Bloomberg BNA Priv Secur Law Rep 13(1581):1–6

[CR26] Schwartz PM, Solove DJ (2014b) Reconciling personal information in the United States and European Union. Calif l Rev 102:877

[CR27] Solymosi R, Buil-Gil D, Ceccato V, Kim E, Jansson U (2023) Privacy challenges in geodata and open data. Area 55(4):456–464

[CR28] Stevens R (2007) Torts and rights. OUP Oxford, Walton Street, Oxford

[CR29] Taylor L (2017) Safety in numbers? Group privacy and big data analytics in the developing world. Group privacy: new challenges of data technologies, Springer, Charm, p 67–82

[CR30] Taylor L, Floridi L, Van der Sloot B (Eds) (2017) Introduction: A New Perspective on Privacy. Group privacy: New challenges of data technologies. Springer

[CR31] UN-GGIM. (2015). Future trends in geospatial information management: the five-to-ten-year vision, Second Edition.

[CR32] Van der Sloot, B (2017) Do groups have a right to protect their group interest in privacy and should they? Peeling the onion of rights and interests protected under article 8 ECHR. Group privacy: new challenges of data technologies 197–224

[CR33] Wang N, Christen M, Hunt M (2021) Ethical considerations associated with “humanitarian drones”: a scoping literature review. Sci Eng Ethics 27(4):51 34342721 10.1007/s11948-021-00327-4 PMC8330183

[CR34] Zwitter A, Gstrein OJ (2020) Big data, privacy and COVID-19–learning from humanitarian expertise in data protection. Journal of International Humanitarian Action 5:1–7 10.1186/s41018-020-00072-6 PMC7232912 38624331

