# Supplementary material for: Examining the applicability of hard data protection law on demographically identifiable information (DII): the case of humanitarian UAV/drone images in Malawi
Source: J Int Humanit Action. 2025 Jul 21;10(1):12. doi: 10.1186/s41018-025-00174-z (PMC12279557; doi:10.1186/s41018-025-00174-z)
Supplement: Supplementary file 1 — Supplementary Material 1. [file 41018_2025_174_MOESM1_ESM.docx]

**Annex 1**. Semi structured interview questions and answers

| Questions to participants | Answers | Percentage |
| --- | --- | --- |
| 1. Do you think UAVs deployed in human settlements and communities for flood or disaster management can collect data which is PII? | YES, strongly agree = 5  YES, agree = 6  Neutral/Not sure = 1  NO, disagree = 5  NO, Strongly disagree = 3 | Positive: 11  Negative: 8  Neutral: 1 |
| 1. Do you think UAVs deployed in human settlements and communities for flood or disaster management can collect data which is DII? | YES, Strongly agree = 10  YES, agree = 10  Neutral/Not sure = /  NO, disagree = /  NO, Strongly disagree = / | Positive: 20  Negative: /  Neutral:/ |
| 1. Do you think high-resolution aerial images or other aerial data collected by means of UAVs are absolutely indispensable for flood or disaster management i.e. without these, there is no other means to produce accurate data for efficient flood or disaster management? | YES, Strongly agree = 4  YES, agree = 11  Neutral/Not sure = 1  NO, disagree = 4  NO, strongly disagree = / | Positive: 15  Negative: 4  Neutral: 1 |
| 1. Do you believe (some) data collected by UAVs of a community could be information which the community residents consider sensitive? | YES, Strongly agree = 9  YES, agree = 9  Neutral/Not sure = 1  NO, disagree = 1  NO, Strongly disagree = | Positive: 18  Negative: 1  Neutral: 1 |
| 1. Do you think high-resolution aerial images or other related data taken via UAVs of a community can tell accurate information about the residents like their main religion, ethnicity, principal occupation, food security levels, economic situation? | YES, Strongly agree = 8  YES, agree = 11  Neutral/Not sure = /  NO, disagree = /  NO, Strongly disagree = 1 | Positive: 19  Negative: 1  Neutral: / |
| 1. Could there be a possibility that data collected using UAVs could, after processing, produce unfair or biased interpretations (probably based on prior data fed into the data processing system i.e. machine learning) and influence decisions which could be detrimental to part of or the entire community? | YES, Strongly agree = 2  YES, agree = 12  Neutral/Not sure = 3  NO, disagree = 3  NO, Strongly disagree = / | Positive: 14  Negative: 3  Neutral: 3 |
| 1. Do you think UAVs deployed to a community for data collection could collect extra data of that community than was originally needed or intended? | YES, Strongly agree = 8  YES, agree = 12  Neutral/Not sure = /  NO, disagree = /  NO, Strongly disagree = / | Positive: 20  Negative: /  Neutral: / |
| 1. Do you think the residents of a community should always be informed about the details of the UAV data collection (that it is taking high-resolution images of them and their community, the purpose, benefits, who is in charge of the project, who the images will be shared with etc) before the UAVs are flown?. | YES, Strongly agree = 16  YES, agree = 4  Neutral/Not sure = /  NO, disagree = /  NO, Strongly disagree = / | Positive: 20  Negative: /  Neutral: / |
| 1. Do you believe high-resolution aerial images of people or their community, or similar aerial data collected through UAVs, could possibly be used by government or the collector organisation for another purpose than the original purpose for which the UAVs were deployed? | YES, Strongly agree = 10  YES, agree = 8  Neutral/Not sure = 1  NO, disagree = 1  NO, Strongly disagree = / | Positive: 18  Negative: 1  Neutral: 1 |
| If this happens, should the people concerned be informed of this change of purpose? | YES, Strongly agree = 11  YES, agree = 6  Neutral/Not sure = 2  NO, disagree = 1  NO, Strongly disagree = | Positive: 17  Negative: 1  Neutral: 2 |
| 1. Do you think there should be a time limit for the collector organisation to store high-resolution aerial images or similar data of a community and/or its residents, after which the images should be deleted? | YES, Strongly agree = /  YES, agree = 3  Neutral/Not sure = 3  NO, disagree = 11  NO, Strongly disagree = 3 | Positive: 3  Negative: 14  Neutral: 3 |
| 1. Should consent of the local residents be sought before carrying out the UAV activity over their community? | YES, Strongly agree = 15  YES, agree = 3  Neutral/Not sure = /  NO, disagree = 2  NO, Strongly disagree = / | Positive: 18  Negative: 2  Neutral: / |
| 1. Should community residents be given the right and power to, at any time, ask the collector organisation to show or present to them all the aerial images or other data it has about them and/or their community? | YES, Strongly agree = 8  YES, agree = 7  Neutral/Not sure = 2  NO, disagree = 3  NO, Strongly disagree = / | Positive: 15  Negative: 3  Neutral: 2 |
| 1. Should residents be given the right and power to, at any time, ask the organisation to delete all aerial images or similar data the organisation has collected about them and/or their community? | YES, Strongly agree = 2  YES, agree = 5  Neutral/Not sure = 2  NO, disagree = 7  NO, Strongly disagree = 4 | Positive: 7  Negative: 2  Neutral: 11 |
| 1. Could UAV-collected data of residents and their communities to be processed, and decisions taken based on the data, solely through automated processing, without involving a human in the organisation at all? | YES, Strongly agree = /  YES, agree = 1  Neutral/Not sure = /  NO, disagree = 13  NO, Strongly disagree = 6 | Positive: 1  Negative: 19  Neutral: / |
| 1. In your opinion, is it necessary for a data collector organisation to have a staff member or office specifically charged with ensuring that data collected about residents and their communities, like high-resolution images, are processed responsibly and in accordance with data protection rules i.e. a Data Protection Officer? | YES, Strongly agree = 14  YES, agree = 6  Neutral/Not sure = /  NO, disagree = /  NO, Strongly disagree = / | Positive: 20  Negative: /  Neutral: / |
| 1. Should the organisation have a responsibility to audit the technology of the UAVs supplied to them by donors to ensure it is data protection compliant (e.g. the donors do not have discreet access to the data)? | YES, Strongly agree = 9  YES, agree = 11  Neutral/Not sure = /  NO, disagree = /  NO, Strongly disagree = / | Positive: 20  Negative: /  Neutral: / |
| 1. If the organisation assigns a third party to collect high-resolution aerial images or similar data of residents and/or their community on its behalf, do you think the organisation should be held liable for any wrongdoings committed by this third party when collecting this data? | YES, Strongly agree = 7  YES, agree = 8  Neutral/Not sure = 1  NO, disagree = 2  NO, Strongly disagree = 2 | Positive: 15  Negative: 4  Neutral: 1 |
| 1. In case there is a security breach and high-resolution images or similar data of residents and their community are hacked and stolen, should the organisation inform the concerned residents of this? | YES, Strongly agree = 5  YES, agree = 11  Neutral/Not sure = 3  NO, disagree = 1  NO, Strongly disagree = | Positive: 16  Negative: 1  Neutral: 3 |
| In case of the above, should the organisation be liable to compensate the residents in cash or kind? | YES, Strongly agree = 1  YES, agree = 7  Neutral/Not sure = 5  NO, disagree = 6  NO, Strongly disagree = 1 | Positive: 8  Negative: 7  Neutral: 5 |
| 1. In your opinion, is it necessary for a prior, detailed impact assessment (to identify and document any data risks and proposed solutions on how to address them) to be done and validated by the organisation before deploying UAVs to a community for data collection? | YES, Strongly agree = 12  YES, agree = 7  Neutral/Not sure = /  NO, disagree = 1  NO, Strongly disagree = / | Positive: 19  Negative: 1  Neutral: / |
